# Burden of occupational cancer in Brazil and federative units, 1990-2019

**DOI:** 10.1590/1980-549720230001

**Published:** 2023-01-06

**Authors:** Viviane Gomes Parreira Dutra, José Henrique Costa Monteiro da Silva, Rafael Tavares Jomar, Henrique Cesar Santejo Silveira, Camila Drumond Muzi, Raphael Mendonça Guimarães

**Affiliations:** I Universidade Estácio de Sá, School of Medicine – Rio de Janeiro (RJ), Brazil.; II Economic Comission for Latin America and the Caribbean – Santiago, Chile.; III Instituto Nacional de Câncer, Hospital Cancer Registry – Rio de Janeiro (RJ), Brazil.; IV Hospital de Câncer de Barretos, Molecular Oncology Research Center – Barretos (SP), Brazil.; V Instituto Nacional de Câncer, Assistance Division — Rio de Janeiro (RJ), Brazil.; VI Fundação Oswaldo Cruz, Sergio Arouca National School of Public Health – Rio de Janeiro (RJ), Brazil.

**Keywords:** Occupational cancer, Neoplasms, Occupational health, Time series analysis, Câncer ocupacional, Neoplasias, Saúde do trabalhador, Análise de séries temporais

## Abstract

**Objective:**

To analyze the spatiotemporal distribution of the burden of occupational cancer in Brazil and federative units between 1990 and 2019.

**Methods:**

Data were extracted from the Global Burden of Disease (GBD) study. Deaths from cancer whose attributable risk factor was occupational carcinogens were considered. Spatial analysis was performed with the first and last years of the series (1990 and 2019). Age-adjusted mortality rates were used to estimate the global Moran’s Index (Moran’s I), and the local indicator of spatial association (LISA) to identify clusters in the country with the respective statistical significance. The occupational cancer mortality rate, adjusted for age, was analyzed based on its trend for Brazil and federative units, in the period between 1990 and 2019.

**Results:**

Between 1990 and 2019, occupational cancer mortality rate showed a decreasing trend (R^2^=0.62; p<0.001) as well as the burden of disease indicator — DALY (R^2^=0.84; p<0.001). However, mortality is increasing in most states, suggesting that a minority of federative units induce the country’s global trend. There is also the development of a spatial pattern of autocorrelation, indicating clusters of states with low mortality and DALY rates in the Northeast and high values in the South of the country.

**Conclusion:**

The overall decreasing trend in the trend of occupational cancer masks the heterogeneity across states. This scenario may be associated with the diversity of economic activities, and suggests a decentralized and equitable plan for occupational cancer surveillance.

## RESUMO

**Objetivo:**

Analisar a carga de câncer relacionado ao trabalho no Brasil e unidades da federação, entre 1990 e 2019.

**Métodos:**

Extraímos os dados do estudo Carga Global de Doenças (GBD). Consideramos as mortes por câncer cujo fator de risco atribuível fossem agentes ocupacionais carcinogênicos. A análise espacial foi realizada com o primeiro e último anos da série (1990 e 2019). As taxas de mortalidade ajustadas por idade foram utilizadas para calcular o índice de Moran global (I de Moran) e o indicador local de associação espacial (LISA). A taxa de mortalidade por câncer relacionado ao trabalho, ajustada por idade, foi analisada com base em sua tendência, para Brasil e unidades da federação, no período entre 1990 e 2019.

**Resultados:**

No período citado, a taxa de mortalidade por câncer relacionado ao trabalho exibiu tendência de decréscimo (R2=0,62; p<0,001), assim como o indicador de carga de doença — DALY (R2=0,84; p<0,001). Contudo, a mortalidade é crescente na maioria dos estados, o que sugere que uma minoria de unidades induz a tendência global do país. Há ainda formação de um padrão espacial de autocorrelação, indicando agrupamentos de estados com baixas taxas de mortalidade e DALY no Nordeste e valores altos no Sul do país.

**Conclusão:**

A tendência global de decréscimo na tendência do câncer relacionado ao trabalho mascara a heterogeneidade entre estados. Esse cenário pode estar associado à diversidade de atividades econômicas e sugere um plano descentralizado e equitativo da vigilância do câncer relacionado ao trabalho.

## INTRODUCTION

Cancer is a noncommunicable chronic disease with worldwide distribution and a still poorly-defined etiology, being considered a public health issue in developed and developing countries^1^. In 2018, global burden estimates showed that 18 million new cases of cancer occurred worldwide, with an adjusted incidence rate of 204.7 cases per 100 thousand men and 175.6 cases per 100 thousand women. Regarding mortality, there were 9.6 million deaths from neoplasms, and cancer was considered the fourth leading cause of premature death (before 70 years of age) in different regions of the world^2^. In Brazil, in 2018, 243,588 deaths from cancer were recorded, with a standardized mortality rate of 109.7 deaths per 100 thousand men and 77.9 deaths per 100 thousand women. Estimates of cancer incidence for the triennium 2020–2022 point to the emergence of more than 600 thousand new cases^3^. Therefore, it is one of the most complex problems that health systems face, considering its epidemiological, social, and economic scope^4^.

The etiology of cancer is multifactorial. Approximately 30% of cancer types are related to environmental factors, which includes occupation. Thus, the increased interest in occupational exposures has grown in the scientific community^5^. One of the reasons for this increased interest is the fact that, over time, estimates show that the occurrence of occupational cancer is close, in magnitude, to deaths due to risk factors (RF) traditionally known and studied^6^.

In 2016, it was estimated that there were 349 thousand deaths and 7.2 million years of life lost due to disability (disability-adjusted life years – DALYs) due to these exposures, with relative variations between regions and ages^7^. Nonetheless, a major challenge in the prevention of occupational cancer is the lack of knowledge of where cancer exposures are taking place and how many workers are affected^8^.

Compared with traditional indices, the DALY indicator combines metrics that incorporate life expectancy and quality of life or disability, in addition to mortality itself. In this sense, changes in public policies can improve not only the measures of death, but also the burden of the disease, which is related to the time that a person can live with a certain disease and how much this can impact their quality of life^7^. For example, diabetes, musculoskeletal disorders, and mental disorders, overall, have low mortality and incidence. However, if these disorders occur early, they can provide a survival with many years of low quality of life to the affected individuals, as they are chronic conditions that directly interfere in the physical, mental, and social capacity of those affected. Thus, the aim of this study was to analyze the burden of occupational cancer in Brazil and federative units between 1990 and 2019.

## METHODS

### Study design

This is a study on the burden of disease associated with occupational cancer, for the period from 1990 to 2019, in Brazil and federative units.

### Data sources

Data were extracted from the Global Burden of Disease (GBD) study (https://www.healthdata.org/gbd/data-visualizations), available from 2022, concerning the 1990–2019 period, at the subnational level for Brazil. Deaths from cancer whose attributable RF was occupational carcinogens were considered for data extraction. The GBD study investigates RF groups. For each of those that were selected, the population attributable risk (PAR) is calculated, which assesses the burden of disease attributable to certain exposures. PAR allows identifying how much of the total risk for a given disease in the general population is due to a certain risk group. With this information, data on cancer deaths whose risk factor is attributed to carcinogenic occupational agents were obtained. Relative risk estimates are based on consistent research results, such as randomized controlled trials, cohort studies, and others, provided they are developed with appropriate methods. In turn, exposure levels and relative risk for each of the listed factors are estimated according to the available literature^9^.

### Inclusion criteria

According to the GDB, the definition of occupational carcinogens includes the proportion of individuals inserted in groups identified as exposed (high and low exposure) to recognized carcinogens (asbestos, arsenic trioxide, acids, benzene, beryllium, cadmium, chromium, diesel, formaldehyde, nickel, polycyclic aromatic hydrocarbons, passive smoking, silica, trichloroethylene), having as reference the distribution of the population in 17 economic sectors^10^. In turn, occupational cancers listed by the GBD include mesothelioma, lung, tracheal and bronchial, laryngeal, ovarian, nasopharyngeal, kidney, esophageal, liver, pancreatic, leukemia, and bladder cancers^11^. It is worth mentioning that the included exposures, as well as cancer locations, are aligned with the classification of the International Agency for Research on Cancer (IARC) and were recently addressed for the Brazilian context^12^.

### Data analysis

#### Spatial analysis

Spatial analysis was conducted with the first and last years of the series (1990 and 2019). Age-adjusted mortality rates were used to estimate the global Moran’s index (Moran’s I) to analyze the global spatial autocorrelation; and the local indicator of spatial association (LISA), to identify local clusters in the country with their statistical significance^13^. Moran’s global and local indices aim to identify patterns of spatial distribution of the presented indicators. The following quadrants are presented to describe the univariate spatial correlation: high-high, low-low, high-low, and low-high. Moreover, in order to observe the spatial pattern for the two indicators concomitantly, a bivariate analysis was conducted and the spatial correlation of DALY and mortality rate was presented.

#### Time series

The occupational cancer mortality rate, adjusted for age, was analyzed based on its trend for Brazil and federative units, in the period between 1990 and 2019. It is assumed that the outcome has no seasonality. Thus, this component of the time series was not included in the model. The analysis followed three steps^14^. The stationarity of the series was evaluated by the Wald-Wolfowitz test. Then, to evaluate the trend direction, the Cox-Stuart test was used. Finally, the trends were analyzed by the polynomial model, whose dependent variable (Y) is represented by age-adjusted mortality rates, and the independent variable (X) is represented by the calendar years, modified from the midpoint of the series (year-centralized variable) to avoid multicollinearity. For choosing the best model, the analysis of residues was performed and the assumption of homoscedasticity and adherence to the normal distribution was evaluated. In addition, the scatter plot and the value of the coefficient of determination (R^2^) were analyzed.

The software R 4.0.0 was used for the analysis of time series, and the GeoDa, version 1.14.0, was used for spatial analysis.

### Ethical considerations

This study is exempt from consideration by the Research Ethics Committee for using secondary, public, and unidentified databases, pursuant to Resolutions No. 466/2012 and 510/2016.

## RESULTS

In Brazil, from 1990 to 2019, the mortality rate from occupational cancer, as well as the DALY indicator, showed a decreasing trend. We can verify this difference between 1990 and 2019 (Table 1). It is worth highlighting that this discrepancy is observable when comparing the rates adjusted for age.

**Table 1:** Burden indicators of occupational cancer. Brazil and federative units, 1990 and 2019.

Region	FU	DALY						Deaths					
		n		Rate				n		Rate			
				Crude		Adjusted				Crude		Adjusted	
		1990	2019	1990	2019	1990	2019	1990	2019	1990	2019	1990	2019
North	Acre	84	439	20.35	47.41	50.88	67.80	3	18	0.80	1.99	2.66	3.30
	Amapá	45	296	16.52	35.03	40.69	50.95	2	11	0.61	1.33	1.84	2.27
	Amazonas	535	1,876	25.81	44.46	63.24	61.65	20	78	0.96	1.85	2.88	2.86
	Pará	1,359	3,573	27.89	38.64	55.72	48.14	47	144	0.97	1.56	2.39	2.13
	Rondônia	282	1,026	25.52	57.75	75.56	62.50	9	42	0.84	2.39	3.96	2.91
	Roraima	64	232	30.53	38.71	92.67	57.15	2	9	1.01	1.52	4.36	2.85
	Tocantins	132	643	14.39	39.15	31.01	43.23	5	27	0.53	1.65	1.59	1.96
Northeast	Alagoas	528	1,585	20.65	43.30	38.09	47.72	21	64	0.83	1.75	1.73	2.04
	Bahia	3,076	9,289	25.38	58.23	41.97	56.50	111	380	0.91	2.38	1.68	2.36
	Ceará	2,093	6,966	32.27	69.42	48.60	68.83	79	310	1.22	3.09	1.95	3.14
	Maranhão	1,258	3,010	24.89	36.01	42.68	44.52	40	130	0.78	1.55	1.52	2.01
	Paraíba	837	2,208	25.61	50.37	36.91	46.91	37	99	1.12	2.26	1.67	2.06
	Pernambuco	1,820	5,477	24.92	54.08	39.19	53.19	73	234	0.99	2.31	1.75	2.39
	Piauí	519	1,304	19.60	35.31	34.02	34.60	19	55	0.72	1.50	1.49	1.47
	Rio Grande do Norte	526	1,860	21.36	49.74	33.01	47.24	22	81	0.90	2.17	1.45	2.07
	Sergipe	338	1,092	22.64	45.33	42.68	46.30	14	44	0.93	1.82	2.05	1.97
Southeast	Espírito Santo	755	2,329	28.68	58.59	48.09	51.23	28	97	1.06	2.45	2.13	2.26
	Minas Gerais	6,501	15,042	40.70	69.35	62.53	56.08	245	657	1.53	3.03	2.76	2.48
	Rio de Janeiro	9,152	13,907	70.03	78.69	87.89	61.07	332	611	2.54	3.46	3.60	2.74
	São Paulo	19,465	35,556	60.82	78.11	89.83	64.02	720	1,579	2.25	3.47	3.92	2.98
South	Paraná	3,422	8,636	39.93	75.82	65.16	62.97	127	378	1.48	3.32	2.93	2.92
	Rio Grande do Sul	9,615	16,354	103.90	144.71	137.17	104.05	363	744	3.92	6.58	5.80	4.77
	Santa Catarina	2,729	7,171	59.96	100.22	100.31	84.01	103	307	2.26	4.29	4.48	3.87
Midwest	Federal District	446	1,242	27.63	41.00	69.37	48.58	15	56	0.93	1.84	3.56	2.87
	Goiás	1,484	3,973	35.84	57.78	62.80	54.14	52	164	1.24	2.38	2.63	2.42
	Mato Grosso	530	1,885	26.69	52.37	58.66	52.71	19	75	0.94	2.08	2.59	2.33
	Mato Grosso do Sul	586	1,747	32.67	61.48	59.49	56.80	21	73	1.18	2.58	2.64	2.55
Brazil		68,180	148,718	45.81	68.64	71.39	61.69	2,526	6,469	1.70	2.99	3.02	2.79

FU: federative units; DALY: disability-adjusted life years.

Source: GBD Study, 2022.

When verifying the crude rates, there is an opposite trend, corroborating the idea that cancer is an outcome dependent on the age structure. There are still notable differences between federative units, which comprise not only the comparison between the extreme years of the historical series, but also the trend. In Table 2 we present the results of trend analysis according to federative units. It should be noted that, although the mortality rate continues to decline, this is not the profile of the federative units. In fact, 15 of them tend to increase and are mostly located in the North and Northeast regions. Except for the states of Pará and Amapá, which did not show a significant trend, all other states presented models with adjustments of variable quality, but statistically significant (R^2^=0.275 [state of Goiás] and R^2^=0.961 [state of Rio Grande do Sul]). Regarding DALY, all federative units showed a significant trend. Contrary to what occurred with the mortality trend, most of them (22) presented a decreasing trend for DALY. Likewise, the models presented adjustments of variable quality (R^2^=0.326 [state of Pará] and R^2^= 0.968 [state of Alagoas]).

**Table 2:** Trend of mortality from occupational cancer. Brazil and federative units, 1990–2019.

Region	Federative Unit	Mortality						
		β_0_	β_1_	β_2_	β_3_	R^2^	p-value	Trend
North	Acre	2.359	0.022	0.002	–	0.894	<0.001	Increasing
	Amapá	2.229	-0.002	-0.001	–	0.060	0.462	NS*
	Amazonas	2.716	0.025	–	–	0.722	<0.001	Increasing
	Pará	1.824	0.003	–	–	0.081	0.143	NS*
	Rondônia	2.649	0.009	–	–	0.174	0.027	Increasing
	Roraima	3.698	0.028	-0.002	–	0.779	<0.000	Decreasing
	Tocantins	1.418	-0.007	0.001	–	0.767	<0.001	Increasing
Northeast	Alagoas	1.403	0.010	0.001	–	0.845	<0.001	Increasing
	Bahia	1.437	0.021	–	–	0.954	<0.001	Increasing
	Ceará	1.995	0.024	-0.001	–	0.887	<0.001	Decreasing
	Maranhão	1.009	0.000	0.002	–	0.701	<0.001	Increasing
	Paraíba	1.669	0.013	–	–	0.649	<0.001	Increasing
	Pernambuco	1.814	0.009	0.001	–	0.654	<0.001	Increasing
	Piauí	1.074	0.012	0.001	–	0.540	<0.001	Increasing
	Rio Grande do Norte	1.546	0.017	0.001	–	0.925	<0.001	Increasing
	Sergipe	1.632	0.002	0.001	–	0.519	<0.001	Increasing
Southeast	Espírito Santo	1.737	-0.018	0.002	–	0.735	<0.001	Increasing
	Minas Gerais	2.212	0.025	0.001	–	0.817	<0.001	Decreasing
	Rio de Janeiro	3.139	-0.039	–	–	0.894	<0.001	Decreasing
	São Paulo	3.494	-0.028	–	–	0.918	<0.001	Decreasing
South	Paraná	2.765	-0.007	0.001	–	0.404	0.002	Increasing
	Rio Grande do Sul	5.496	-0.063	–	–	0.961	<0.001	Decreasing
	Santa Catarina	3.980	-0.025	–	–	0.751	<0.001	Decreasing
Midwest	Federal District	3.299	-0.016	-0.002	–	0.822	<0.001	Decreasing
	Goiás	2.072	-0.003	0.001	–	0.275	0.018	Increasing
	Mato Grosso	2.459	0.017	-0.001	–	0.491	<0.001	Decreasing
	Mato Grosso do Sul	2.729	0.005	-0.001	–	0.391	0.002	Decreasing
Brazil		2.713	-0.008	–	–	0.617	<0.001	Decreasing

Region	Federative Unit	DALY						
		β_0_	β_1_	β_2_	β_3_	R^2^	p-value	Trend
North	Acre	50.539	0.516	0.027	–	0.902	<0.001	Increasing
	Amapá	44.294	-0.448	0.012	0.004	0.645	<0.001	Increasing
	Amazonas	59.297	0.842	-0.012	-0.004	0.753	<0.001	Decreasing
	Pará	42.097	0.210	-0.018	-0.002	0.326	0.022	Decreasing
	Rondônia	54.082	0.768	0.014	-0.005	0.681	<0.001	Decreasing
	Roraima	66.743	-0.848	–	–	0.920	<0.001	Decreasing
	Tocantins	29.815	0.164	0.016	–	0.836	<0.001	Decreasing
Northeast	Alagoas	33.458	0.646	0.011	-0.002	0.968	<0.001	Decreasing
	Bahia	33.983	0.479	–	–	0.938	<0.001	Increasing
	Ceará	46.356	0.411	-0.031	–	0.862	<0.001	Decreasing
	Maranhão	24.186	0.355	0.041	-0.004	0.929	<0.001	Decreasing
	Paraíba	38.834	0.919	0.016	-0.004	0.917	<0.001	Decreasing
	Pernambuco	41.972	0.245	–	–	0.628	<0.001	Increasing
	Piauí	24.446	0.654	0.009	-0.003	0.793	<0.001	Decreasing
	Rio Grande do Norte	36.336	0.428	–	–	0.955	<0.001	Increasing
	Sergipe	38.691	0.109	0.015	–	0.445	<0.001	Increasing
Southeast	Espírito Santo	40.040	0.044	0.016	-0.002	0.814	<0.001	Decreasing
	Minas Gerais	49.822	0.474	0.011	-0.004	0.741	<0.001	Decreasing
	Rio de Janeiro	70.104	-0.995	–	–	0.949	<0.001	Decreasing
	São Paulo	73.514	-0.638	–	–	0.921	<0.001	Decreasing
South	Rio Grande do Sul	116.905	-1.469	–	–	0.961	<0.001	Decreasing
	Paraná	58.093	0.204	-0.002	-0.002	0.692	<0.001	Decreasing
	Santa Catarina	82.482	-0.434	–	–	0.761	<0.001	Decreasing
Midwest	Federal District	54.618	-0.626	–	–	0.868	<0.001	Decreasing
	Goiás	44.291	0.215	0.011	-0.002	0.409	0.005	Decreasing
	Mato Grosso	54.611	0.942	-0.032	-0.005	0.702	<0.001	Decreasing
	Mato Grosso do Sul	58.350	0.097	-0.019	–	0.502	<0.001	Decreasing
Brazil		59.623	-0.272	–	–	0.844	<0.001	Decreasing

DALY: disability-adjusted life years.

* NS: not significant.

Source: GBD Study, 2022.

**Figure 1:**
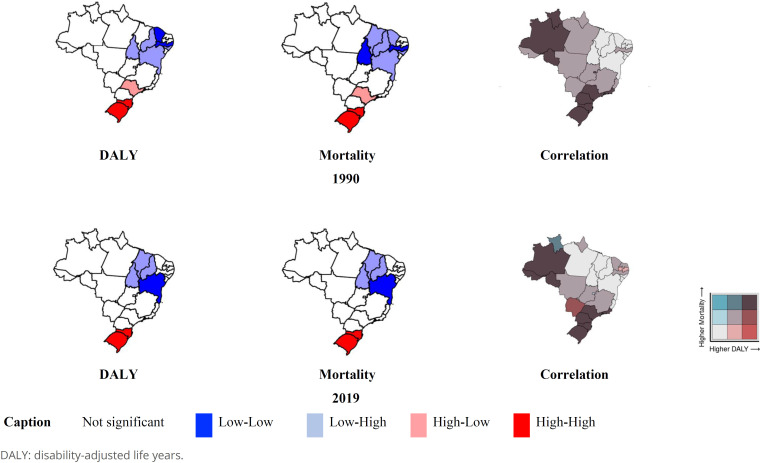
Spatial autocorrelation of occupational cancer burden indicators in Brazil, including bivariate spatial correlation, according to federative unit. Brazil, 1990 and 2019.

Source: GBD, 2022.

In Figure 1 we present the results of the analysis of the local spatial autocorrelation (Moran’s LISA). Moran’s I showed statistical significance (p-value<0.001) for global spatial autocorrelation of occupational cancer in Brazil, both for mortality and DALY. By employing the LISA method, we observed autocorrelation at the local level and spatial clusters. There are high-high clusters between states of the Southern region, whereas the low-low and low-high clusters are located in the Northeast region.

Finally, still in Figure 1, we analyzed the bivariate spatial correlation between mortality and DALY due to occupational cancer in Brazil in the two studied periods, by federative unit. The general results show spatial heterogeneity over the period. Regarding regional inequality, high mortality and high DALY correlation groups persist in the Southern regions and in the Northern Amazon region of the country. Conversely, federative units with correlation between low mortality and low DALY are grouped in the Northeast region.

## DISCUSSION

Mortality rates and DALY due to occupational cancer tended to decrease in Brazil throughout the period. It is worth mentioning that, over the three decades, there has been an increase in metabolic risks. In addition, it should be noted that the study on global burden of diseases has a hierarchy of risk factors. Occupational risks are at the first level, along with other behavioral and metabolic factors. However, age-standardized overall mortality for some groups of chronic conditions has decreased, as documented in the GBD 2019 Study. This paradox has been attributed to the effect of access to health care, social determinants of health, cohort effects, and other behavioral risks, including changes in occupational health surveillance practices^15^.

Although decreasing in the country, we observed heterogeneity in the trends for the federative units. This evidence allows us highlighting the need for decentralized policies, under the sole command of the Federal Government, providing a level-equitable approach throughout the country^16^.

First, it is important to recognize that Brazil is a country of continental size, whose productive activities are widely spread throughout the geographic space. Activities of the primary, secondary, and tertiary sectors are developed in the country’s territory. Particularly the industry, which is part of the secondary sector, has a higher concentration in the South and Southeast regions, especially the metropolitan region of São Paulo. Conversely, the primary sector of Brazil has prominence in the following regions: Midwest, with a more qualified standard in agribusiness; North, with plant and mineral extraction; and Northeast, with standard of monoculture agricultural production and low-tech mining^17^.

Regarding locations, recent estimates show that, even in more conservative scenarios, between 3.9 and 4.2% of all incident cases of cancer can be attributed to occupational exposure, with most of them being mesothelioma-type cancers, non-melanoma skin cancer, lung, female breast, and urinary bladder^18^. The study conducted by Purdue^19^ showed that the potential impact of work on the genesis of cancer ranged from 2 to 8% (men, 3–14%; women, 1–2%). Furthermore, in more specific studies, the fraction attributable to occupational cancer identified in the literature can reach over 30%, as is the case of lung cancer^5^. In fact, these locations are included in the study, along with others that GBD identifies as work-related^11^.

It is likely that the increase in mortality rates in more than half of the federative units reflects the improvement in the quality of the completion of Death Certificates, especially in the North and Northeast regions of the country^20^; and that the increase in these rates among women and the decrease among men is due to the greater favoritism of the entry and increase of women in the labor market from the 1970s onwards, when there was an expansion of the economy, increasing urbanization and industrialization at an accelerated pace^21^. Thereafter, women started sharing with men the burden of occupational exposure to carcinogens.

Our results demonstrated a high-high spatial autocorrelation of occupational cancer both for mortality and DALY in the states of Santa Catarina and Rio Grande do Sul. A reasonable explanation for these findings is that the Southern region is one of the most industrialized in the country, especially in the processing of primary products and in the production of parts and metallurgy^22^, and these industries may be involved in occupational exposure to carcinogens.

Occupational cancers are largely preventable^23^. Nevertheless, it is worth emphasizing that the patterns of disease screening vary according to the characteristics of the occupation. It is known, for example, that the adherence of workers from small companies is lower than that of workers from large organizations as well as occupational activities that require lower level of education. It is also known that, when adjusted, these associations cease to exist, which suggests, therefore, that the type of occupation is a proxy for socioeconomic status and access to healthcare services^24^. These findings underscore the need for innovative public health strategies to improve cancer screening in vulnerable populations. As the decentralization of the productive process ends up exposing a population that, in itself, is already vulnerable, the social and political context of labor relations must be recognized, especially the fact that most developing countries lack political mechanisms to ensure the protection of workers^25^.

Moreover, it is remarkable that the detection of occupational cancer is partly difficult due to characteristics typical of natural history. Occupational exposures are often of low intensity and long duration, increasing the latency period to the disease^26^. It is quite common to discover it only after the work activity itself has been completed. Although levels of many exposures have been reduced in recent years, the long latency means that past high exposures will continue to result in substantial numbers in the near future^27^. Thus, despite controversies about the accuracy of quantitative estimates, there is a certain consensus that occupational cancer tends to be concentrated among relatively small groups of people, but who have a high risk in the development of the disease^28^.

It is noteworthy that all these estimates about the burden of occupational cancer are somewhat vulnerable to biases that may lead to underestimation of occupational burden, such as the exclusion of possible or probable carcinogens (following the IARC classification), the exclusion of cancer locations that are not emphatically described in the literature as being related to work, or a gap in the evidence of association with substances that have not yet been studied regarding carcinogenic potential^29^.

In fact, there are opportunities to revitalize comprehensive global cancer control policies, incorporating primary interventions against environmental and occupational carcinogens^30^. In Brazil, the Brazilian Ministry of Health, in 2018, developed the *Atlas of Work-related Cancer in Brazil* to identify occupational and environmental factors that pose a risk for cancer, promoting an improvement in occupational health surveillance. This atlas provides the analysis of 18 cancer locations that are work-related. Spatial descriptions of mortality data are presented, as well as age-cohort-period analyses for all locations, based on standardized and corrected mortality rates, considering the fraction of these cancers that is attributed to occupation^31^. This product was developed in the creation of a list that includes carcinogenic agents, established or probable, present in the productive processes of some economic activities in Brazil, aiming at implementing monitoring actions and, ultimately, devising an action plan for the control of occupational cancer^32^.

More recently, the Brazilian Ministry of Health published a new edition of the Atlas, seeking to promote a national surveillance of occupational cancer^33^. However, the update was limited to presenting regionalized analyses of mortality by locations associated with work in its entirety. First, the adopted analysis strategy makes a comparison with the virtually limited attributable fraction, as it does not associate occupational exposure with the occurrence of cancer, but death due to this cancer. This event, nevertheless, is permeated by a number of other circumstances such as timely access to diagnosis and treatment. In addition, the Atlas analysis does not discriminate, in the historical series of mortality, what fraction of these deaths is attributable to work, either precisely, year by year, or in the trend. This measure is dependent on other characteristics besides the outcome such as the prevalence of occupational exposure to carcinogens. Only the historical series of the locations of cancers with some evidence of being work-related is observed, without necessarily having a causality. Hence, it would not be possible, by analyzing the Atlas, to evaluate the volume of cancer that could be avoided with the cessation of exposure. While the evidence provided by the Atlas is very limited to sectoral action on occupational cancer control — which was its original purpose —, the surveillance effort to make this field a priority should be recognized.

Our findings illustrate the repercussions of occupational exposure on cancer burden as one of the effects of work on health. Despite the fact that cancer prevention requires cessation of exposure to individual risk factors, such as smoking and consumption of processed foods, it is necessary to understand the contextual effect of exposure, especially those attributed to vulnerable populations such as occupational exposure^34^.

Therefore, in addition to the evident implication for the quality of life of workers, it is worth highlighting that productivity losses associated with cancer in the workplace are significant. At the same time that we used secondary data standardized by international methodology in this research, we sought to promote the visibility of this important public health issue, already stimulated by the Brazilian Ministry of Health in recent years.

All in all, the detection of occupational risks should be a priority in occupational health surveillance, reinforcing the need to develop strategies for preventing and controlling occupational cancer in Brazil from the perspective of public health and workers’ health.
